# Real-world patient characteristics and use of disease-modifying anti-rheumatic drugs in patients with rheumatoid arthritis: a cross-national study

**DOI:** 10.1007/s10067-022-06478-4

**Published:** 2022-12-19

**Authors:** Ylenia Ingrasciotta, Yinzhu Jin, Saveria S. Foti, Joan E. Landon, Michele Tari, Francesco Mattace-Raso, Seoyoung C. Kim, Gianluca Trifirò

**Affiliations:** 1 Department of Diagnostics and Public Health, University of Verona, Verona, Italy; 2 Academic Spin-Off “INSPIRE-Innovative Solutions For Medical Prediction And Big Data Integration In Real World Setting”-Azienda Ospedaliera Universitaria “G. Martino”, Messina, Italy; 3 Department of Internal Medicine, Erasmus MC University–Medical Center, Rotterdam, The Netherlands; 4 Division of Pharmacoepidemiology and Pharmacoeconomics, Brigham and Women’s Hospital and Harvard Medical School, Boston, MA USA; 5 Caserta Local Health Unit, Caserta, Italy; 6 Division of Rheumatology, Inflammation, and Immunity, Brigham and Women’s Hospital and Harvard Medical School, Boston, MA USA

**Keywords:** Biologics, Claims database, Disease-modifying anti-rheumatic drugs, Real-world data, Rheumatoid arthritis

## Abstract

**Introduction:**

Rheumatoid arthritis (RA) is associated with significant morbidity and economic burden. This study aimed to compare baseline characteristics and patterns of anti-inflammatory drug use and disease-modifying anti-rheumatic drug (DMARD) use among patients with RA in Southern Italy versus the United States.

**Method:**

Using Caserta Local Health Unit (Italy) and Optum’s de-identified Clinformatics® Data Mart (United States) claims databases, patients with ≥ 2 diagnosis codes for RA during the study period (Caserta: 2010–2018; Optum: 2010–2019) were identified. Baseline patient characteristics, as well as proportion of RA patients untreated/treated with NSAIDs/glucocorticoids/conventional DMARDs (csDMARDs)/biological/targeted synthetic DMARDs (b/tsDMARDs) during the first year of follow-up, and the proportion of RA patients with ≥ 1 switch/add-on between the first and the second year of follow-up, were calculated. These analyses were then stratified by age group (< 65; ≥ 65).

**Results:**

A total of 9227 RA patients from Caserta and 195,951 from Optum databases were identified (two-thirds were females). During the first year of follow-up, 45.9% RA patients from Optum versus 79.9% from Caserta were exclusively treated with NSAIDs/glucocorticoids; 17.2% versus 11.3% from Optum and Caserta, respectively, were treated with csDMARDs, mostly methotrexate or hydroxychloroquine in both cohorts. Compared to 0.6% of RA patients from Caserta, 3.2% of the Optum cohort received ≥ 1 b/tsDMARD dispensing. Moreover, 61,655 (33.7%) patients from Optum cohort remained untreated compared to 748 (8.3%) patients from the Caserta cohort. The subgroup analyses stratified by age showed that 42,989 (39.8%) of elderly RA patients were untreated compared to 18,666 (24.9%) young adult RA patients in Optum during the first year of follow-up. Moreover, a higher proportion of young adult RA patients was treated with b/tsDMARDs, with and without csDMARDs, compared to elderly RA patients (Optum_<65_: 6.4%; Optum_≥65_: 1.0%; *P*-value < 0.001; Caserta_<65_: 0.8%; Caserta_≥65_: 0.1%; *P*-value < 0.001). Among RA patients untreated during the first year after ID, 41.2% and 48.4% RA patients from Caserta and Optum, respectively, received NSAIDs, glucocorticoids, and cs/b/tsDMARDs within the second year of follow-up. Stratifying the analysis by age groups, 50.6% of untreated young RA patients received study drug dispensing within the second year of follow-up, compared to only 36.7% of elderly RA patients in Optum. Interestingly, more young adult RA patients treated with csDMARDs during the first year after ID received a therapy escalation to b/tsDMARD within the second year after ID in both cohorts, compared to elderly RA patients (Optum_<65_: 7.8%; Optum_≥65_: 1.8%; Caserta_<65_: 3.2%; Casert_a≥65_: 0.6%).

**Conclusions:**

Most of RA patients, with heterogeneous baseline characteristics in Optum and Caserta cohorts, were treated with anti-inflammatory/csDMARDs rather than bDMARDs/tsDMARDs during the first year post-diagnosis, especially in elderly RA patients, suggesting a need for better understanding and dealing with barriers in the use of these agents for RA patients.

**Table:** 

Key Points
*• Substantial heterogeneity in baseline characteristics and access to bDMARD or tsDMARD drugs between RA patients from the United States and Italy exists.*
*• Most of RA patients seem to be treated with anti-inflammatory/csDMARD drugs rather than bDMARD/tsDMARD drugs during the first year post-diagnosis.*
*• RA treatment escalation is less frequent in old RA patients than in young adult RA patients.*
*• An appropriate use of DMARDs should be considered to achieve RA disease remission or low disease activity.*

**Supplementary Information:**

The online version contains supplementary material available at 10.1007/s10067-022-06478-4.

## Introduction

Rheumatoid arthritis (RA) is a chronic systemic inflammatory disease that affects the joints, connective tissues, muscle, tendons, and fibrous tissue and is associated with significant morbidity and economic burden [1–4]. The estimated prevalence of RA worldwide varies between 0.3 and 1% and is more common in women and in developed countries [5]. In the United States (US), RA affects approximately 1.3 million adults [6, 7]. In Italy, the RA prevalence is 0.3–0.7%, confirming a higher prevalence in women than in men [8]. RA commonly affects patients aged 30–50 years old [9], and in patients aged above 60, the prevalence is equal to 2% [10].

Elderly RA patients present frequently comorbidity such as cognitive impairment, depression, and frailty [11]. High incidence of comorbidities and drug-related adverse effects in elderly patients also raise therapeutic challenges for the disease management and to achieve a clinical remission of the disease [12, 13].

Evidence from the literature indicates that, despite available treatments, several unmet needs still exist with regard to RA management [14, 15]. Patients with RA experience substantial levels of pain and are not satisfied with their levels of physical functioning even with ongoing treatment [16]. Currently, the main therapeutic target for RA patients is achieving clinical remission, with low disease activity as the best possible alternative [17], to prevent functional impairment and disability [18, 19]. According to national and international guidelines and recommendations [17, 20, 21], several treatments for RA are available: glucocorticoids or non-steroidal anti-inflammatory drugs (NSAIDs), conventional disease-modifying anti-rheumatic drugs (csDMARDs), targeted synthetic DMARDs (tsDMARDs), and biological DMARDs (bDMARDs).

According to the disease severity, the use of these agents aims at controlling systemic inflammation to slow or prevent the disease progression. Methotrexate is considered the standard of care for RA; in patients with at least one contraindication such as severe hepatic or renal impairment, serious, acute, or chronic infections, and other contraindications or intolerance to methotrexate, leflunomide, or sulfasalazine could be considered as options in the first-line strategy of treatment. Moreover, if the treatment target is not achieved with the first csDMARD strategy, addition/switch to a tsDMARD or a bDMARD is recommended [17, 20, 21].

Over the past 20 years, the management of RA has radically changed. The choice of therapies, which were previously mostly based on csDMARDs, has expanded with the marketing of bDMARDs and, more recently, with the new class of tsDMARDs [22]. In particular, the introduction of bDMARDs has revolutionized treatments for RA, with a substantial positive effect on the quality of care of RA patients who suffer from moderate-to-severe disease or who have failed to improve with other medications [23]. However, due to the high cost of these drugs, heterogeneity in access to bDMARDs in RA patients across Europe has been observed [24].

In 2013, the European Medicine Agency (EMA) approved the first infliximab biosimilar, while the Food and Drug Administration (FDA) did in 2016. In general, biosimilars provide a 20–30% purchase cost reduction in comparison to the reference product, representing a valid cost containing strategy [25]; although health resources are limited, it is widely shared that innovative medicines should be made available to all citizens; as new biologic drugs are expensive, correct management strategies must be implemented.

Although the use of biologics has revolutionized the RA therapeutic landscape, leading to major changes in therapeutic targets, concerns about decreased efficacy due to immune senescence and a low benefit-risk profile in the elderly have led to a relative underutilization of biologics [26]. A rapidly ageing population and increasing rates of RA make the paucity of data in older adults with RA an increasingly important clinical issue.

Moreover, since the efficacy and safety of b/tsDMARDs have been thoroughly investigated in randomized clinical trials (RCTs) [27], real-world studies exploring the pattern of use of RA treatments in routine rheumatology practice considering unselected patients potentially representing the entire spectrum of disease severity are needed. The main objective of this study is to evaluate and compare the baseline characteristics and the pattern of real-world use of drugs (e.g., anti-inflammatory drugs and DMARDs) for the treatment of RA in Southern Italy versus the United States. The second aim of this study is to compare the pattern of real-world use of drugs for the treatment of RA young adult versus elderly RA patients in both countries.

## Materials and methods

### Data sources

This is a retrospective, cross-national cohort study. Data were extracted from Caserta Local Health Unit (LHU)-Italy and Optum’s de-identified Clinformatics® Data Mart-United States claims databases (DBs), covering 1.1 million and 53.3 million individuals, respectively, from January 2010 to September 2019 (Caserta: Jan 2010–Dec 2018). In particular, collected Italian data included demographics, outpatient pharmacy, hospital discharge database, requests for outpatient diagnostic tests and specialist’s visits, exemptions from healthcare service co-payment, and emergency department visit databases. All databases can be linked through an anonymous subject identifier. In addition, general practitioner’s prescriptions (from Arianna database) with related indication for use as well as electronic therapeutic plans (filled by the specialist and including information on drug prescribed, indication for use, drug dosages, and therapy duration) and results of diagnostic tests are collected in Caserta database. The Caserta LHU claims and General Practitioner Arianna databases have been shown to provide accurate and reliable information for pharmacoepidemiological research, as documented elsewhere [28–32]. In Caserta LHU DB, drug dispensing is coded using the Anatomical Therapeutic Chemical (ATC) classification system or specific Italian market authorization code (AIC), while indications for use and causes of hospitalizations are coded using the International Classification of Diseases, 9th revision, clinical modification (ICD9-CM). In Optum DB, drug dispensing is coded using generic names or J/Q codes if applicable, while indications for use and causes of hospitalizations are coded using ICD9-CM or ICD-10 codes.

Moreover, in Italy, biological drugs are fully reimbursed by the National Health Service (NHS) and for each biologic drug prescription, specialists have to fill a therapeutic plan, which indicates the exact drug name, number of dispensed packages, dosing regimen, and indication for use. Electronic therapeutic plans were available in the Caserta LHUs. These data can be linked through unique and anonymous patient identifiers to other claims databases, which contain several types of information, including causes of hospitalization and reasons for healthcare service co-payment exemptions.

Optum Clinformatics® Data Mart (CDM) is derived from a database of de-identified administrative health claims for members of large commercial and Medicare Advantage health plans. The database includes approximately 17–19 million annual covered lives, for a total of over 62 million unique lives over a 13-year period (1/2007 through 12/2020). Clinformatics® Data Mart is statistically de-identified under the Expert Determination method consistent with HIPAA and managed according to Optum® customer data use agreements. CDM administrative claims submitted for payment by providers and pharmacies are verified, adjudicated, and de-identified prior to inclusion. This data, including patient-level enrollment information, is derived from claims submitted for all medical and pharmacy healthcare services with information related to healthcare costs and resource utilization. The population is geographically diverse, spanning all 50 states. Optum de-identified CDM contains longitudinal information on medical and pharmacy claims from a number of different managed care plans, including hospitalizations, outpatient visits, procedures, and pharmacy dispensing. All the medical/pharmacy claims through Optum insurance are recorded in the database as long as the patients were still enrolled in the insurance. As reported for Caserta LHU claims and General Practitioner Arianna databases, Optum Clinformatics® Data Mart has been shown to provide accurate and reliable information for pharmacoepidemiological research, as documented elsewhere [33–36].

### Study population

All patients aged ≥ 18 years with at least two RA diagnoses separated by ≥ 7 days but < 365 days were eligible for the study cohort. The date of the second RA diagnosis was defined as the index date (ID), and patients were required to have at least 1-year pre- and post-index continuous enrollment in their databases to ensure comprehensive availability of data on their healthcare use over this period [36–38]. In the Optum database, RA diagnoses were identified based on RA ICD-9 codes (714.xx) or ICD-10 codes (M05.xx, M06.xx, M08.xx, M12.xx) from inpatient or outpatient medical claims. In the Caserta database, RA diagnoses were identified based on RA ICD-9 codes (714.xx) from discharge diagnosis or emergency department visits or electronic therapeutic plans or from the General Practitioner database (i.e., Arianna database) which can be linked through anonymous subject identifier with claims databases. All patients with any csDMARD, bDMARD, or tsDMARD dispensing any time prior to the first RA diagnosis date were excluded. The identification criteria for the study cohort are shown in Online Resource 1.

### Exposure assessment

All the following drug classes were included: anti-inflammatory drugs (e.g., NSAIDs and glucocorticoids), csDMARDs (e.g., methotrexate, sulfasalazine, leflunomide, chloroquine, hydroxychloroquine, cyclosporine, azathioprine, auranofin, and sodium aurotiosulfate), bDMARDs, both originators and biosimilars (e.g., etanercept, adalimumab, infliximab, certolizumab pegol, golimumab, anakinra, abatacept, sarilumab, tocilizumab, and rituximab), and tsDMARDs (e.g., tofacitinib and baricitinib). Upadacitinib was not included because it was approved by EMA and by FDA in 2019. Online Resource 2 shows all the included drugs for this study.

### Data analysis

In each cohort, the following baseline patient characteristics were assessed: sex, age (categorized as follows: 18–44, 45–64, 65–79, ≥ 80, mean ± standard deviation) at ID, index year, geographic area of patients, comorbidities (e.g., hypertension, diabetes mellitus, chronic pulmonary disease, lipid metabolism disorders, chronic renal failure, liver disease, heart failure, ischemic heart disease, malignancy, smoking, obesity, psoriasis, and inflammatory bowel diseases) evaluated within 1 year prior to ID, number of unique prescription drugs based on generic names (categorized as 0, 1, 2, 3–5, 6–10, > 10) evaluated within 1 year prior to ID, and concomitant drugs (e.g., traditional NSAIDs, COX-2 inhibitors, opioids, antidepressant drugs, antihypertensive drugs, insulin and oral hypoglycemic agents, and lipid lowering agents) evaluated within 1 year prior to ID.

The proportion of RA patients treated or untreated within 1 year after ID in each cohort was calculated. Patients were categorized as follows:

- Untreated patients: patients without any study drug dispensing;
- Exclusive NSAID users: patients with at least one NSAID dispensing AND no dispensing of oral/parenteral glucocorticoids/bDMARD/csDMARD/tsDMARD;
- Glucocorticoid (± NSAID) users: patients with at least one oral/parenteral glucocorticoid dispensing AND no dispensing of csDMARD/bDMARD/tsDMARD;
- csDMARD (± glucocorticoid ± NSAID) users: patients with at least one csDMARD dispensing AND no dispensing of bDMARD/tsDMARD; or
- bDMARD/tsDMARD (± NSAID ± glucocorticoid ± csDMARD) users: patients with at least one bDMARD or tsDMARD dispensing.

Moreover, the proportion of each treatment type among RA patients, after excluding those who were never treated during the follow-up, was calculated. This analysis was then stratified by active substance, distinguishing between originator and biosimilar bDMARDs. Moreover, the proportion of RA patients with at least one switch/add-on between the first and the second year post-ID was calculated. Only RA patients with at least 2 years post-index continuous enrollment in the database were included.

### Subgroup analysis

Subgroup analyses of the proportion of RA patients untreated or treated within 1 year after ID in each cohort and of the proportion of RA patients with at least one switch/add-on between the first and the second year post-ID were conducted according to age (< 65; ≥ 65).

### Statistical analysis

Descriptive statistics were used for the aforementioned baseline variables. For comparisons between the two cohorts, a standardized mean difference (SMD) greater than 0.1 was considered as a sign of imbalance [39]. Statistical analyses were performed using SAS 9.2 (SAS Institute, Cary, NC, USA).

## Results

**Fig. 1:**
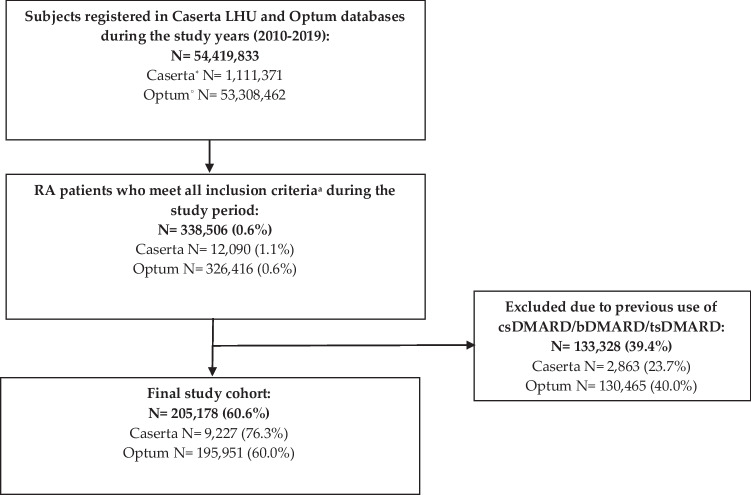
Flow chart of the study cohort. Legend: LHU, Local Health Unit; csDMARDs, conventional disease-modifying anti-rheumatic drugs; bDMARDs, biological disease-modifying anti-rheumatic drugs; tsDMARDs, targeted synthetic disease-modifying anti-rheumatic drugs. *Data available until December 2018. °Data available until September 2019. Patients: (a) age ≥ 18 years; (b) ≥ 2 diagnoses of RA, separated by ≥ 7 days but < 365 days; (c) ≥ 1 year pre-index and 1-year post-index date continuous enrollment in their databases

During the study period, 195,951 and 9227 subjects with a diagnosis of RA were identified from Optum and Caserta databases, respectively (Fig. 1). RA prevalence was higher in Caserta (1.1%) than in Optum (0.6%). Of these, more than two-thirds were female patients in both cohorts [Optum: *N* = 133,605 (68.2%); Caserta: *N* = 6117 (66.3%); SMD = 0.0408]. RA patients from Optum were older than those from Caserta (mean age ± SD: 66.8 ± 14.2 years in Optum vs. 57.1 ± 16.1 years in Caserta; SMD = 0.6788) (Table 1). In particular, 119,026 (60.7%) and 3203 (34.7%) RA patients were aged 65 years or over, in Optum and Caserta, respectively.

**Table 1:** Baseline characteristics of the study cohort

	Optum *N* = 195,951	Caserta *N* = 9227	SMD (*d*)
Sex — *N* (%)			
Male	62,346 (31.8)	3110 (33.7)	0.0408
Female	133,605 (68.2)	6117 (66.3)	
Mean age ± SD — year	66.8 ± 14.2	57.1 ± 16.1	0.6788
Age — *N* (%)			
18–44	18,377 (9.4)	2184 (23.7)	0.3921
45–64	58,538 (29.9)	3840 (41.6)	0.2459
65–79	84,433 (43.1)	2506 (27.2)	0.3377
≥ 80	34,593 (17.6)	697 (7.5)	0.3084
Geographic area of patients — *N* (%)			
Northeast	27,167 (13.9)	-	-
South	87,936 (44.9)		
Midwest	39,045 (19.9)		
West	41,803 (21.3)		
Index year — *N* (%)			
2010	14,449 (7.4)	489 (5.3)	0.0861
2011	13,330 (6.8)	1338 (14.5)	0.2515
2012	13,330 (6.8)	1623 (17.6)	0.3345
2013	12,955 (6.6)	1589 (17.2)	0.3318
2014	12,518 (6.4)	1349 (14.6)	0.2699
2015	18,515 (9.4)	899 (9.7)	0.0102
2016	36,639 (18.7)	1087 (11.8)	0.1928
2017	41,071 (21.0)	853 (9.3)	0.3307
2018	33,144 (16.9)	-	-
Comorbidities — *N* (%)^a^			
Hypertension	131,949 (67.3)	4350 (47.1)	0.4293
Diabetes mellitus	56,861 (29.0)	1117 (12.1)	0.3589
Chronic pulmonary disease	45,651 (23.3)	1886 (20.4)	0.0687
Hyperlipidemia	115,589 (59.0)	1656 (17.9)	0.8431
Chronic renal failure	17,921 (9.1)	144 (1.6)	0.2657
Liver diseases	13,433 (6.9)	126 (1.4)	0.2209
Heart failure	20,334 (10.4)	192 (2.1)	0.2768
Ischemic heart disease	39,307 (20.1)	938 (10.2)	0.2495
Malignancy	16,072 (8.2)	455 (4.9)	0.1213
Smoking	35,639 (18.2)	778 (8.4)	0.2568
Obesity	31,445 (16.0)	199 (2.2)	0.3837
Inflammatory bowel disease	2452 (1.2)	214 (2.3)	0.099
Psoriasis	3745 (1.9)	181 (2.0)	0.0073
Previous use of any medications — mean ± SD	9.7 ± 7.6	9.8 ± 6.3	0.0133
Previous use of any medications — *N* (%)^a^			
0	25,224 (12.9)	53 (0.6)	0.5056
1	6521 (3.3)	166 (1.8)	0.0952
2	6261 (3.2)	404 (4.3)	0.0579
3–5	25,862 (13.1)	1907 (20.7)	0.2038
6–10	53,085 (27.1)	3135 (34.0)	0.1502
> 10	78,998 (40.3)	3562 (38.6)	0.0347
Concomitant drugs — *N* (%)^a^			
Traditional NSAIDs	79,690 (40.7)	7531 (81.6)	0.8397
COX-2 inhibitors	10,318 (5.3)	2242 (24.3)	0.8014
Opioids	98,619 (50.3)	1312 (14.2)	0.7305
Antidepressant drugs	49,347 (25.2)	1205 (13.1)	0.2812
Antihypertensives	135,834 (69.3)	4741 (51.4)	0.3866
Insulin and oral hypoglycemic agents	33,122 (16.9)	1168 (12.7)	0.1126
Lipid lowering agents	73,127 (37.3)	2197 (23.8)	0.2806

Legend: *SMD*, standardized mean difference; *SD*, standard deviation; *NSAIDs*, non-steroidal anti-inflammatory drugs. ^a^Evaluated within 1 year prior to ID

In general, compared to the Caserta cohort, a higher proportion of the Optum cohort had comorbidities at baseline (80.0% vs. 63.2%). Specifically, hypertension [Optum: *N* = 131,949 (66.3%); Caserta: *N* = 4350 (47.1%); SMD = 0.4294] and hyperlipidemia [Optum: *N* = 115,589 (59.0%); Caserta: *N* = 1656 (17.9%); SMD = 0.8431] were the two most common comorbidities in both cohorts. In both cohorts, less than 2% of RA patients had other autoimmune disorders for which bDMARDs might be indicated (e.g., inflammatory bowel diseases and psoriasis). Interestingly, 40.2% of patients from both cohorts had received more than 10 drugs during the 1-year period prior to the ID. Half of RA patients from Optum had received at least one dispensing for opioids, compared to 14% of RA patients from Caserta (SMD = 0.7305). Contrarily, 7531 (81.6%) and 2242 (24.3%) in the Caserta cohort had received traditional NSAIDs and COX-2 inhibitors, respectively, versus 79,690 (40.7%) and 10,318 (5.3%) in the Optum cohort (SMD_traditional NSAIDs_ = 0.8397; SMD_COX-2 inhibitors_ = 0.8014).

### DMARD treatment patterns

**Fig. 2:**
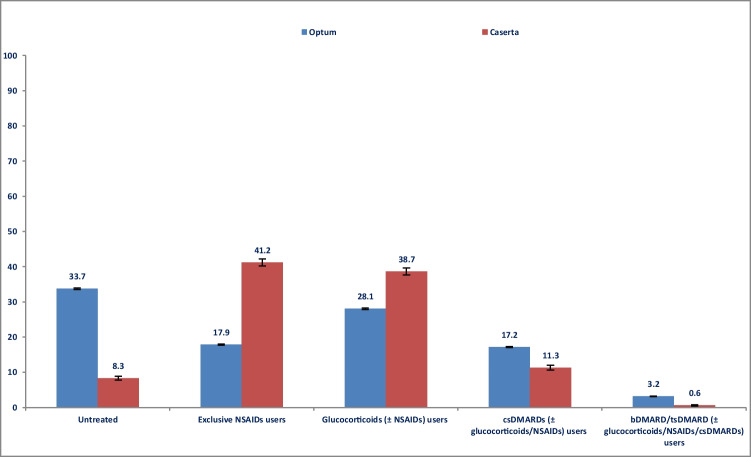
Frequency (%) of treatment lines within the first year after ID. Legend: DMARD, disease-modifying anti-rheumatic drug; csDMARD, conventional synthetic disease-modifying anti-rheumatic drug; tsDMARD, targeted synthetic disease-modifying anti-rheumatic drug; bDMARD, biological disease-modifying anti-rheumatic drug

During the first year of follow-up, one-third (*N* = 61,655; 33.7%) of RA patients from Optum were untreated with NSAIDs, glucocorticoids, or any DMARDs, compared to 748 (8.3%) RA patients from Caserta (*P*-value < 0.001). Among treated patients, almost half (84,036; 45.9%) of RA patients from Optum versus more than two-thirds (*N* = 7199; 79.9%) from Caserta received NSAIDs/glucocorticoids dispensing (*P*-value < 0.001), but they did not receive specific RA treatments (e.g., csDMARDs, bDMARDs, or tsDMARDs); 17.2% of patients from Optum versus 11.3% of patients from Caserta were treated with csDMARDs (*P*-value < 0.001) (Fig. 2), mostly methotrexate or hydroxychloroquine in both cohorts. No sodium aurothiosulfate users were identified in both cohorts (Online Resource 3). Compared to 3.2% of RA patients from Optum, only 0.6% of RA patients from Caserta had at least one bDMARD/tsDMARD dispensing, with and without csDMARDs (*P*-value < 0.001) (Fig. 2). The most frequently used bDMARD was the adalimumab originator (Optum: 1.4%; Caserta: 0.2%; *P*-value < 0.001), followed by the etanercept originator (Optum: 1.1%; Caserta: 0.1%; *P*-value < 0.001). In both cohorts, no patients used anakinra, adalimumab biosimilars, or e rituximab biosimilars; no users of sarilumab were identified in Caserta (Online Resource 3). We found no tsDMARD users in Caserta versus 226 tsDMARD users (224 tofacitinib and 2 baricitinib) in Optum.

**Fig. 3:**
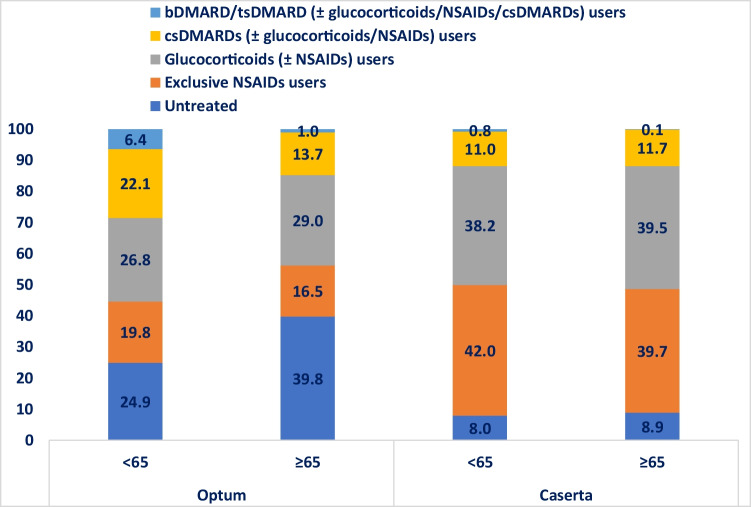
Frequency (%) of treatment lines within the first year after ID, stratified by age group. Legend: DMARD, disease-modifying anti-rheumatic drug; csDMARD, conventional synthetic disease-modifying anti-rheumatic drug; tsDMARD, targeted synthetic disease-modifying anti-rheumatic drug; bDMARD, biological disease-modifying anti-rheumatic drug

The subgroup analysis stratified by age showed that 42,989 (39.8%) of elderly RA patients were untreated compared to 18,666 (24.9%) young adult RA patients in Optum (*P*-value < 0.001) (Fig. 3). Specifically, 14,851 (13.7%) elderly RA patients versus 16,553 (22.1%) young adult RA patients from Optum received csDMARDs during the first year after ID (*P*-value < 0.001). Concerning the use of csDMARDs from the Caserta cohort, no statistically significant differences were observed in the two age groups compared. Regarding the use of bDMARDs/tsDMARDs, a higher proportion of young adult RA patients was treated with bDMARDs/tsDMARDs, with and without csDMARDs, compared to elderly RA patients (Optum < 65: 6.4%; Optum ≥ 65: 1.0%; *P*-value < 0.001; Caserta < 65: 0.8%; Caserta ≥ 65: 0.1%; *P*-value < 0.001).

**Fig. 4:**
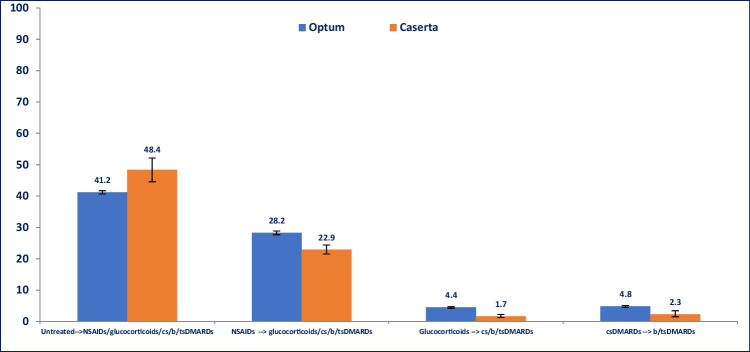
Proportion (%) of RA patients with at least one switch/add-on between the first and the second year after ID. Legend: DMARD, disease-modifying anti-rheumatic drug; csDMARD, conventional synthetic disease-modifying anti-rheumatic drug; tsDMARD, targeted synthetic disease-modifying anti-rheumatic drug; bDMARD, biological disease-modifying anti-rheumatic drug

Among untreated RA patients during the first year after ID, 41.2% from Optum and 48.4% from Caserta received at least one study drugs dispensing within the second year of follow-up (*P*-value < 0.001) (Fig. 4).

In general, almost two-thirds (63.3%) of US elderly RA patients versus 49.4% of young adult RA patients continued to be untreated between the first and the second year after ID (*P*-value < 0.001).

**Fig. 5:**
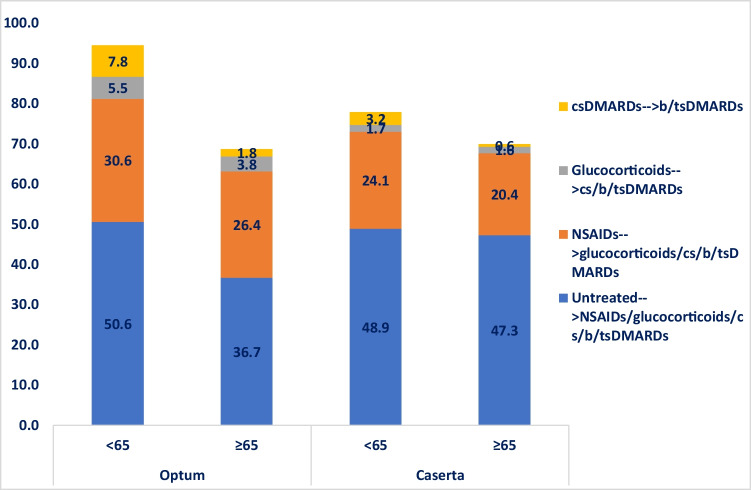
Proportion (%) of RA patients with at least one switch/add-on between the first and the second year after ID, stratified by age. Legend: DMARD, disease-modifying anti-rheumatic drug; csDMARD, conventional synthetic disease-modifying anti-rheumatic drug; tsDMARD, targeted synthetic disease-modifying anti-rheumatic drug; bDMARD, biological disease-modifying anti-rheumatic drug

Stratifying the analysis by age groups, more than half (50.6%) of untreated young RA patients during the first year after ID received study drug dispensing within the second year of follow-up, compared to only 36.7% of elderly RA patients in Optum (*P*-value < 0.001). Among untreated patients from Caserta, no statistically significant differences were observed in the two compared age groups (*P*-value: 0.689) (Fig. 5). Interestingly, more young adult RA patients treated with csDMARDs during the first year after ID received a therapy escalation to b/tsDMARD within the second year after ID in both cohorts, compared to elderly RA patients (Optum < 65: 7.8%; Optum ≥ 65: 1.8%; *P*-value < 0.001; Caserta < 65: 3.2%; Caserta ≥ 65: 0.6%; *P*-value: 0.012).

## Discussion

This large retrospective cross-national population-based cohort study investigated the baseline characteristics and the pattern of use of different pharmacological treatment lines (anti-inflammatory drugs, csDMARDs, bDMARDs, and tsDMARDs) in patients with RA from the US and Italy over the 10-year study period. Our data about RA prevalence suggest that it was higher in Caserta than in Optum, but in line with prevalence reported in literature [5, 7, 8]. As expected, the distribution by sex showed a female/male ratio equal to 2:1 in both cohorts. In general, a higher proportion of RA patients from Optum had comorbidities at baseline, and they were older than RA patients from Caserta. Specifically, hypertension and hyperlipidemia were the two most common comorbidities, followed by obstructive pulmonary disease, in both cohorts. This is in line with a prospective Swedish study [40] as well as a cohort study using a commercial and Medicare claims database with national beneficiaries [36], showing that 47.1% and 39.3% of RA patients had history of hypertension, followed by 31.9% patients with chronic obstructive pulmonary disease.

In both our cohorts, less than 2% of RA patients had history of other autoimmune disorders for which bDMARDs might be indicated (e.g., inflammatory bowel diseases and psoriasis), as reported by Jin et al. [36]. This is also due by the exclusion of all RA patients with at least one csDMARD, bDMARD, or tsDMARD dispensing any time prior to the first RA diagnosis date.

On average, RA patients from both cohorts had received more than 10 drugs within 1 year prior to the ID. Half of RA patients from Optum had received at least one dispensing for opioids, compared to 14% of RA patients from Caserta. It is known that abuse of opioids for the treatment of chronic pain is very common in the US. Recent years have seen an “opioid crisis” take place in the US, with widespread overuse and misuse of opioids, leading to a large number of overdose-related deaths [30, 41]. Zamora-Legoff et al., in a population-based study including RA patients from the Rochester Epidemiology Project (REP), a special record-linkage system that records all inpatient and outpatient encounters among the residents of Olmsted County, Minnesota, showed that over a third of RA patients used opioids, and in more than a tenth, the use was chronic [42]. Contrarily, our findings showed a higher use of traditional NSAIDs and COX-2 inhibitors at baseline among RA patients from Caserta than those in the US. The highest use of NSAIDs in Italy was confirmed by an Italian population-based study evaluating the clinical characteristics of elderly analgesic users in Caserta LHU and the frequency of potentially inappropriate analgesic use [30]. The study showed that, among 94,820 elderly persons receiving at least one analgesic drug, 36.6% were incident NSAID users, while 13.2% were incident weak opioid users and 8.1% were incident strong opioid users. Specifically, 9.2% of all elderly analgesic users were considered to have an inappropriate prescription for the NSAIDs (ketorolac or indomethacin) [30].

During the first year of follow-up, one-third of RA patients from Optum seem to be untreated with either NSAIDs, glucocorticoids, or any DMARDs, compared to 8% of RA patients from Caserta. Specifically, almost 40% of US elderly RA patients were untreated compared to 25% of US young adult RA patients during the first year after ID, while no statistically significant differences were observed in the two age groups compared in the Caserta cohort. Moreover, our results showed that, overall, among untreated RA patients, almost half of patients from both study cohorts received at least one study drug dispensing within the second year of follow-up; however, almost two-thirds of US elderly RA patients versus half of young adult RA patients continued to be untreated between the first and the second year after ID. This is in line with a previous study, showing that more than 50% of adults aged 45 years or older with some forms of arthritis remain untreated, despite many of them experiencing severe symptoms and poor physical function [43]. Nevertheless, an exploratory analysis showed that the proportion of untreated RA patients decreased to 6% in Optum and 2% in Caserta within 3 years after ID (data not shown). Regarding those treated, almost half of RA patients from Optum versus more than two-thirds of RA patients from Caserta received NSAIDs/glucocorticoids dispensing, but they did not receive RA-specific DMARD treatments. Among csDMARDs, mostly methotrexate and hydroxychloroquine were used in both cohorts. This is in line with national and international guidelines and recommendations [17, 20, 21]. Methotrexate remains the mainstay 1st-line DMARD in RA; not only is it an efficacious csDMARD by itself but it is also the basis for combination therapies, either with glucocorticoids or with other csDMARDs, bDMARDs, or tsDMARDs. The European Alliance of Associations for Rheumatology (EULAR) guidelines recommend that in patients with a contraindication to methotrexate (or early intolerance), leflunomide or sulfasalazine should be considered as part of the (first) treatment strategy [17, 20]. However, our results showed a low use of leflunomide and sulfasalazine in both countries, compared to hydroxychloroquine. However, EULAR guidelines state that antimalarials, and especially hydroxychloroquine, have a limited role, mainly reserved for patients with mild RA [17] given the only weak clinical and no structural efficacy of hydroxychloroquine [44].

According to the guidelines, bDMARDs/tsDMARDs represent a 2nd line of therapy usually reserved for patients who have failed or have contraindications to csDMARDs [17, 20, 21]. Although RA treatment has made major advances over the past few decades, especially with the introduction of biologics as a treatment option for RA patients, most of the patients in our study were found to be initially treated with anti-inflammatory drugs or csDMARDs rather than bDMARDs. This may be due to the patients in the study having had less severe RA or a state of low disease activity that warranted no treatment with biologic agents. It could also be that patients may still have been kept on csDMARDs despite not achieving remission or low disease activity as recommended in the RA guidelines [17, 20]. Given that claims databases do not collect clinical data on effectiveness or disease activity, we were not able to evaluate these hypotheses.

However, our results are confirmed by an Italian retrospective observational study using claims databases from Veneto, Marche, Abruzzo, Apulia, and Calabria Regions [45]. The mentioned study showed that, as a first treatment, 5% of RA patient received bDMARDs versus 52% were not treated with DMARDs and received no treatment at all or only NSAIDs/glucocorticoids versus 43% of RA patients receiving csDMARDs (83% of csDMARD users continued with the same category of DMARDs during the follow-up).

Similar evidence from the US showed that only 2.6% of RA patients initiated b/tsDMARD treatment within 1 year of diagnosis [46], confirming the low use of bDMARDs/tsDMARDs in our two cohorts, especially in elderly patients from US. A recent retrospective, cohort study using the US Corrona RA registry showed that 54% of RA patients with persistent moderate-to-high disease activity after 6 months of treatment with a csDMARD drug did not receive their therapy escalation. Of the patients who completed a visit at 3–9 months after the index date, treatment advancement occurred in 29% of the patients, with 71% having no change. Dose escalation of the csDMARD, initiation of another csDMARD, and initiation of a bDMARD occurred in 13%, 8%, and 10% of patients of the total population [47].

Our results showed that treatment escalation was less frequent in old RA patients than in young adult RA patients. Different studies have suggested that old RA patients may be less aggressively treated than they should be [10, 26, 48, 49]. The Ruban study reported that despite higher disease activity at diagnosis, elderly-onset RA (EORA) patients were less likely to receive combination DMARD therapies or biologic agents compared with young-onset RA (YORA) patients, even though these drugs (biologics in particular) have been shown to have similar efficacy in older and younger individuals [49]. Howard et al. showed that time to first biologic DMARD is strongly associated with age. The ≥ 75 s were more likely to be on less intensive therapies compared to the < 65 s (csDMARD monotherapy or steroid alone, versus csDMARD combination therapy or bDMARD).

This may in part be due to access, as public payers take longer than private payers to recognize criteria for use and issue approval of advanced therapeutic agents. Indeed, the access to bDMARDs/tsDMARDs still represents an insight. In Italy, although bDMARDs/tsDMARDs are fully reimbursed by the NHS, the access barrier is due to the guidelines, which recommend these high-cost treatments if the treatment target is not achieved with the csDMARD strategy. On the contrary, in the US, the access barrier to these high-cost treatments could be explained by the high median out-of-pocket cost (e.g., $ 40 for bDMARDs and $ 50 for tsDMARDs).

Our study showed that the most frequently used bDMARD was the adalimumab originator, followed by the etanercept originator. A very low proportion of RA patients received infliximab biosimilars, while no users of adalimumab biosimilar and rituximab biosimilar in both cohorts were identified. The first reimbursement approval by the Italian NHS was in July 2017 for rituximab biosimilar and August 2018 for adalimumab biosimilar. Concerning rituximab biosimilar dispensing, it may not be traced in Caserta DB because it was rarely used by Caserta LHU hospitals. Adalimumab biosimilar dispensing may not be traced in the Caserta database because the mean/median times lag between the Italian Drug Agency (AIFA) and Campania Drug Formulary Committee approval could reach some months. In the US, even though five adalimumab and two rituximab biosimilars have been approved by FDA, they were not marketed during the study period [50]. No users of anakinra in both cohorts as well as no users of sarilumab (Italian reimbursement at the end of 2018) in Caserta were identified during the study years. Anakinra was approved for the treatment of moderate‐severe RA but not generally used for RA anymore due to its lower effectiveness when compared to studies using other biologic therapies [51]. Concerning tsDMARDs (i.e., tofacitinib and baricitinib), less than 0.2% of RA users from Optum versus no users in Caserta were identified because of recent reimbursement approval of these drugs.

The main strength of this population-based study is the large size and generalizability of the study cohort and the availability of the claims data from the US as well as a Local Health Unit from Southern Italy for the past decade. We acknowledge some limitations of our study, due to the descriptive nature of the analysis, based on data collected through administrative claims databases. However, real-world observational studies provide evidence on how specific drugs are used in the market and what impact they have in the long-term on the already limited health resources. This is in contrast with randomized controlled trials where data are limited to the experimental conditions of the trial design, and where results may not translate fully to the real-world [52–56]. Second, we cannot exclude a potential misclassification of RA patients from the US, thus resulting in a high proportion of untreated RA patients during the first year of follow-up. However, we defined our cohort selection based on previous studies [36–38] and we required all Optum patients to have continuous insurance enrollment during the study period to avoid misclassification due to insurance switching. Furthermore, the traceability of some pharmacy claims, such as NSAIDs/glucocorticoids, might not have been captured by the two databases because they are used as over-the-counter drugs or privately purchased; consequently, the proportion of untreated RA patients could be overestimated; an exploratory analysis was carried using a database provided by IMS Health on pharmacy sales data for all pharmacies in Caserta LHU in the years 2014–2018. Prescription data from IMS are aggregate prescription-level data through which it is possible to distinguish between units of drugs dispensed through the NHS and those purchased privately by citizens. This analysis showed that more than half of NSAIDs and glucocorticoids packages acquired in community pharmacies were bought privately and could not have been captured by the NHS administrative drug dispensing databases. On the contrary, csDMARDs, bDMARDs, and tsDMARDs were fully reimbursed and then traceable. Third, another limitation is represented by the lack of data in the administrative claims databases on clinical outcome measures, such as the effectiveness of treatment, disease severity, and other potential confounders, that could have influenced our results. Finally, our findings from Caserta may not be fully representative of those in the whole Italian general population. However, the applied methodology and the Caserta LHU claims database as well as the Arianna database have been shown to provide accurate and reliable information for pharmacoepidemiological research, as documented elsewhere [28–31].

## Conclusions

In conclusion, our study showed substantial heterogeneity in baseline characteristics and access to bDMARD or tsDMARD drugs between RA patients from the United States and Italy. Most RA patients in our study were treated with anti-inflammatory drugs or csDMARDs, especially elderly, rather than bDMARDs or tsDMARDs during the first year post-diagnosis, suggesting a need for better understanding and dealing with barriers in the use of these agents for diagnosed RA patients. In particular, regardless of age, appropriate use of DMARDs should be considered to achieve RA disease remission or low disease activity. With the increasing spectrum of therapeutic options and the new information on existing drugs, this study could be helpful to provide insights into the management of RA patients in clinical practice.

## Supplementary Information

Below is the link to the electronic supplementary material.

Online Resource 1. Depiction of the study cohort identification criteria. Legend: Dx: RA diagnosis; MARD: Disease-Modifying Anti-Rheumatic Drug (JPG 193 KB)

Online Resource 2. Study drugs approved for the treatment of RA. Legend: DMARD: Disease-Modifying Anti-Rheumatic Drug; csDMARD: Conventional Synthetic Disease-Modifying Anti-Rheumatic Drug; tsDMARD: Targeted Synthetic Disease-Modifying Anti-Rheumatic Drug; bDMARD: Biological Disease-Modifying Anti-Rheumatic Drug; AIC= Italian market authorization code; ATC= anatomical therapeutic chemical classification system (PDF 318 KB)

Online Resource 3. Frequency (%) of different compound within the first year after ID. Legend: csDMARD: Conventional Synthetic Disease- Modifying Anti-Rheumatic Drug; tsDMARD: Targeted Synthetic Disease Modifying Anti-Rheumatic Drug; bDMARD: Biological Disease Modifying Anti-Rheumatic Drug. Note: Only compounds with proportions ≥0.05% were showed (PDF 188 KB)

## Data Availability

Concerning Caserta Local Health Unit database, fully anonymized dataset is available only upon request to the corresponding author, as there is an agreement between the University of Messina and the data provider (Caserta Local Health Unit) not to share the data publicly.
