# Supplementary figures and images for: Real-world patient characteristics and use of disease-modifying anti-rheumatic drugs in patients with rheumatoid arthritis: a cross-national study
Source: Clin Rheumatol. 2022 Dec 19;42(4):1047–59. doi: 10.1007/s10067-022-06478-4 (PMC10017582; doi:10.1007/s10067-022-06478-4)

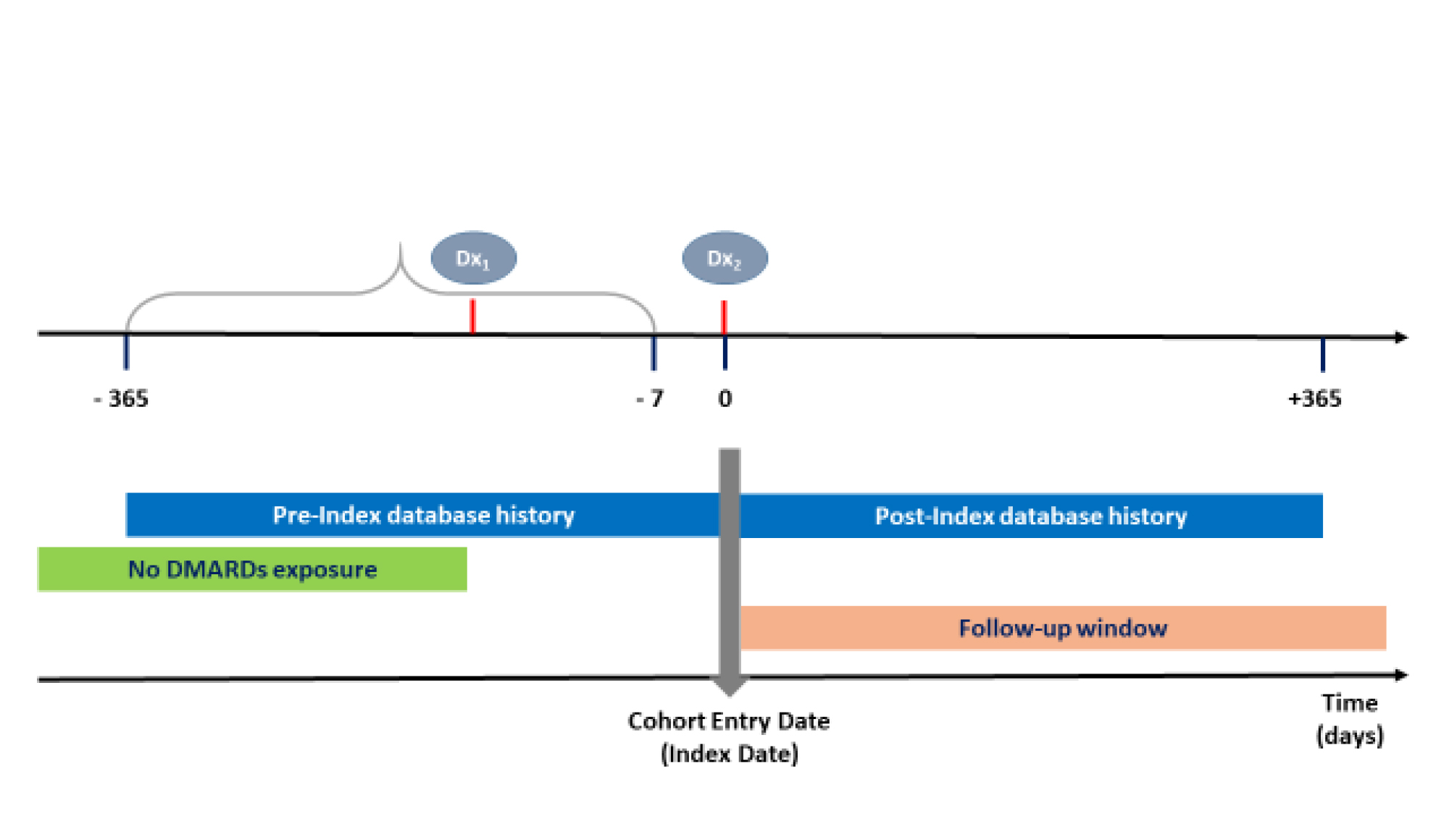

Supplement: Supplementary file 1 — Online Resource 1. Depiction of the study cohort identification criteria. Legend: Dx: RA diagnosis; MARD: Disease-Modifying Anti-Rheumatic Drug (JPG 193 KB) [file 10067_2022_6478_MOESM1_ESM.jpg]

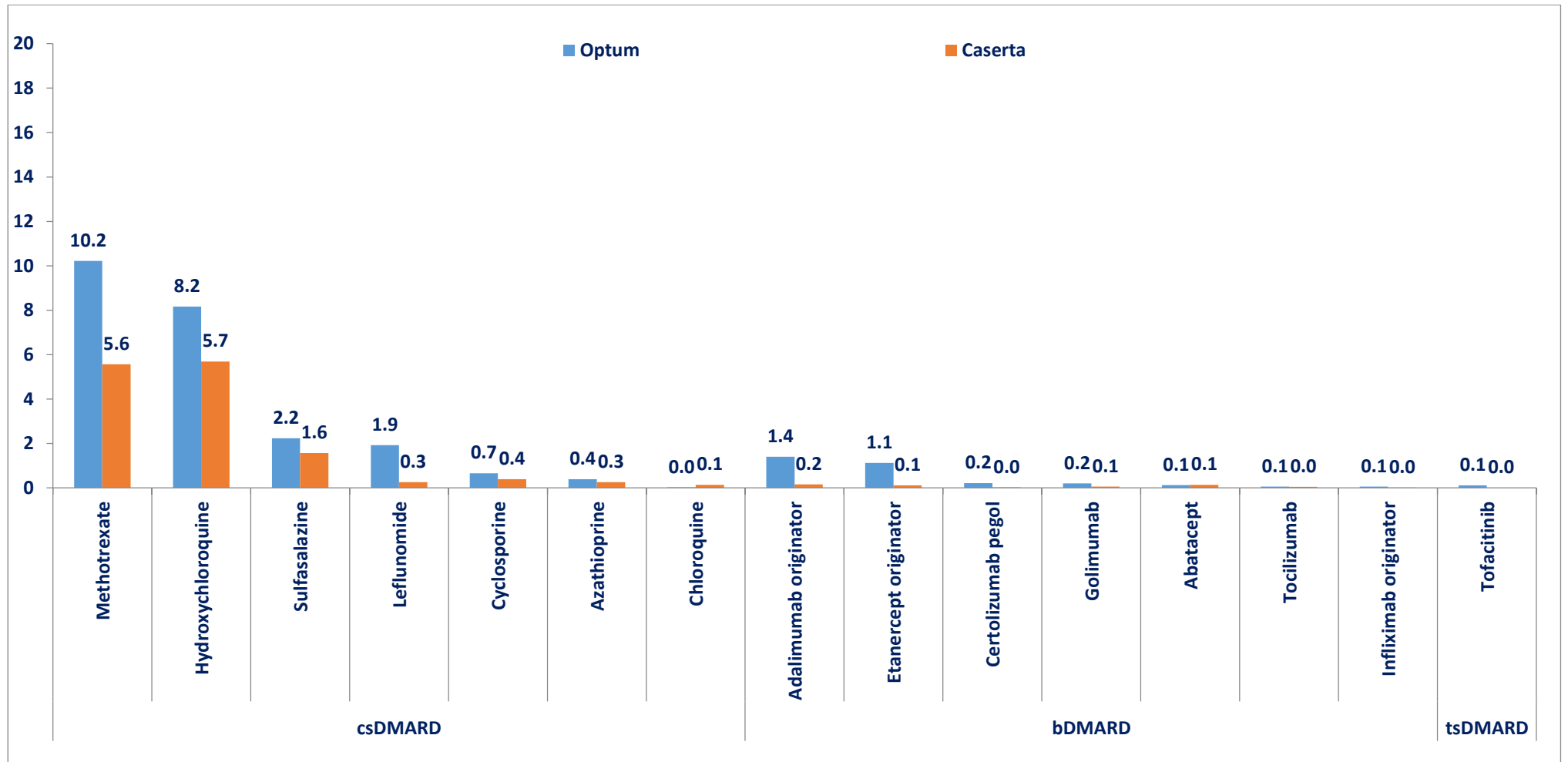

Supplement: Supplementary file 3 — Online Resource 3. Frequency (%) of different compound within the first year after ID. Legend: csDMARD: Conventional Synthetic Disease- Modifying Anti-Rheumatic Drug; tsDMARD: Targeted Synthetic Disease Modifying Anti-Rheumatic Drug; bDMARD: Biological Disease Modifying Anti-Rheumatic Drug. Note: Only compounds with proportions ≥0.05% were showed (PDF 188 KB) [file 10067_2022_6478_MOESM3_ESM.pdf]
