# Supplementary material for: Real-world patient characteristics and use of disease-modifying anti-rheumatic drugs in patients with rheumatoid arthritis: a cross-national study
Source: Clin Rheumatol. 2022 Dec 19;42(4):1047–59. doi: 10.1007/s10067-022-06478-4 (PMC10017582; doi:10.1007/s10067-022-06478-4)
Supplement: Supplementary file 2 — Online Resource 2. Study drugs approved for the treatment of RA. Legend: DMARD: Disease-Modifying Anti-Rheumatic Drug; csDMARD: Conventional Synthetic Disease-Modifying Anti-Rheumatic Drug; tsDMARD: Targeted Synthetic Disease-Modifying Anti-Rheumatic Drug; bDMARD: Biological Disease-Modifying Anti-Rheumatic Drug; AIC= Italian market authorization code; ATC= anatomical therapeutic chemical classification system (PDF 318 KB) [file 10067_2022_6478_MOESM2_ESM.pdf]

Drug group	Generic names	Brand name	Type of bDMARD (Originator/biosimilar)	ATC code	AIC code	J/Q codes if applicable
**Anti-inflammatory drugs**						
**Glucocorticoids**	-	-	-	H02AB%%	-	-
**NSAIDs**	-	-	-	M01A%%%	-	-
**csDMARD**						
	Methotrexate	-	-	L01BA01	-	J8610, J9250, J9260
				L04AX03	-	
	Leflunomide	-	-	L04AA13	-	-
	Sulfasalazine	-	-	A07EC01	-	-
	Chloroquine	-	-	P01BA01	-	-
	Hydroxychloroquine	-	-	P01BA02	-	-
	Cyclosporine	-	-	L04AD01	-	-
	Azathioprine	-	-	L04AX01	-	-
	Auranofin	-	-	M01CB03	-	-
	Sodium aurothiosulfate	-	-	M01CB02	-	-
**bDMARD**						
**TNF-alpha inhibitors**	Infliximab	REMICADE	Originator	L04AB02	034528012	J1745
		INFLECTRA	Biosimilar		043010*	Q5103
		FLIXABI	Biosimilar		044892*	Q5104, Q5102, Q5109
		REMSIMA	Biosimilar		042942*	
		ZESSLY	Biosimilar		046635*	
	Etanercept	ENBREL	Originator	L04AB01	034675*(excl. 034675215)	J1438
		BENEPALI	Biosimilar		044691*	-
		ERELZI	Biosimilar		045451*	-
	Adalimumab	HUMIRA	Originator	L04AB04	035946*	J0135
		HULIO	Biosimilar		047088*	-
		IMRALDI	Biosimilar		045616*	-

		HYRIMOZ	Biosimilar		046889*	-
		AMGEVITA	Biosimilar		045317*	-
		IDACIO	Biosimilar		047805*	-
		HALIMATOZ	Biosimilar		046888*	-
	Certolizumab pegol	CIMZIA	Originator	L04AB05	-	J0717, J0718, C9249
	Golimumab	SIMPONI	Originator	L04AB06	-	J1602
**Interleukin inhibitors**	Anakinra	KINERET	Originator	L04AC03	-	-
	Sarilumab	KEVZARA	Originator	L04AC14	-	-
	Tocilizumab	ROACTEMRA	Originator	L04AC07	-	J3262
**Selective costimulation modulator**	Abatacept	ORENCIA	Originator	L04AA24	-	J0129
**Monoclonal anti-CD20 antibody**	Rituximab	MABTHERA/RITUXAN	Originator	L01XC02	033315019	J9310, J9312
					033315021	-
		TRUXIMA	Biosimilar		045266*	Q5115
		RIXATHON	Biosimilar		045450*	
**JAK inhibitors/tsDMARD**						
	Tofacitinib	-	-	L04AA29	-	-
	Baricitinib	-	-	L04AA37	-	-
